# Combined Use of Sodium Bentonite and Recycled Aggregates in Concrete: Microstructural Characterization and Mechanical Performance

**DOI:** 10.3390/ma19204278

**Published:** 2026-10-10

**Authors:** Hugo Morales-Beltrán, Edelmira Gálvez, Eleazar Salinas-Rodríguez, Manuel Saldana, Norman Toro, María del Pilar Gutiérrez-Amador, Williams Leiva

**Affiliations:** 1 Academic Area of Earth Sciences and Materials, Institute of Basic Sciences and Engineering, Autonomous University of the State of Hidalgo, Highway Pachuca-Tulancingo km. 4.5, Mineral de la Reforma, Pachuca 42184, Mexico; mo198072@uaeh.edu.mx; 2 Departamento de Ingeniería Metalúrgica y Minas, Universidad Católica del Norte, Antofagasta 1270709, Chile; egalvez@unc.cl; 3 Faculty of Engineering and Architecture, Universidad Arturo Prat, Iquique 1110939, Chile; notoro@unap.cl; 4 Department of Chemical Engineering and Mineral Processing, University of Antofagasta, Antofagasta 1270300, Chile; 5 Apan High School, Autonomous University of the State of Hidalgo, Highway Apan-Calpulalpan km. 8, Apan 43920, Mexico; amadorg@uaeh.edu.mx; 6 Facultad de Ingeniería, Universidad San Sebastián, Sede Concepción, Concepción 4030000, Chile; williams.leiva@uss.cl

**Keywords:** recycled concrete, construction and demolition waste, sodium bentonite, microstructure, X-ray diffraction, microstructural characterization, non-structural concrete

## Abstract

**Highlights:**

- The 100% CDW aggregate control achieved 19.81 MPa (202.0 kg/cm^2^, 101.0% of target) vs. 15.35 MPa (156.6 kg/cm^2^, 78.3% of target) for 5 wt% sodium bentonite at 28 days, representing a 22.5% reduction relative to control.
- XRD identified tobermorite, calcite, ettringite, and quartz; SEM showed acicular and dense globular morphologies consistent with hydrated phases; and EDS showed homogeneous Ca, Si, and Al distribution. As the control was not microstructurally characterized, features cannot be attributed solely to bentonite.
- Bentonite characterization: SG 2.70; LOI 10.10 ± 0.76% at 750 °C for 45 min; particle size < 75 µm; XRD, montmorillonite/illite/quartz/albite.
- Strength level falls within 9.81–19.61 MPa specified in the 2025 Mexico City General Unit Price Schedule (Tabulador) for blinding layers, slabs, and low-demand floors.

**Abstract:**

The reuse of construction and demolition waste (CDW) and supplementary aluminosilicate materials can reduce the environmental impact of concrete. This study evaluated two formulations produced with fine and coarse recycled CDW aggregates: a control with 0 wt% sodium bentonite and an experimental mixture with 5 wt% sodium bentonite as a partial Portland cement replacement (ACI 211.1-91, target 19.61 MPa at 28 days). Two independent control batches achieved 19.833 MPa (202.24 kg/cm^2^) and 19.788 MPa (201.78 kg/cm^2^) (mean 19.810 MPa, 101.01% of target). The 5 wt% bentonite mixture achieved a mean of 15.353 MPa (156.55 kg/cm^2^, SD 0.060 MPa, CV 0.391%, 95% CI, 15.290–15.416 MPa, *n* = 6), equivalent to 78.29% of the target strength and 22.50% lower than the control mean. The six specimens containing 5 wt% bentonite were technical replicates obtained from a single batch, whereas control values correspond to two independent batches (one specimen each); therefore, confidence intervals are presented descriptively. Characterization of the 5 wt% formulation at 28 days was performed by XDR, SEM, and EDS. XRD identified calcite, ettringite, quartz, and signals compatible with calcium silicate hydrate (C-S-H) products and tobermorite. SEM reveals a heterogeneous matrix with acicular morphologies compatible with C-S-H and densified regions. EDS showed O 54.8 wt%, Ca 27.8 wt%, Si 10.2 wt%, and Al 2.8 wt% with heterogeneous distribution of Ca, Si, and Al without marked segregation at the observation scale. Characterization was limited to the 5 wt% formulation at 28 days; therefore, observed phases and morphologies cannot be attributed exclusively to bentonite.

## 1. Introduction

The construction industry is one of the largest consumers of raw mineral materials and one of the major generators of solid waste worldwide. In this context, construction and demolition waste (CDW) represents a significant fraction of urban waste, posing environmental challenges associated with its final disposal and the need to implement sustainable valorization strategies [1]. One of the most studied alternatives is the use of CDW as recycled aggregates in concrete production, which can reduce the extraction of natural aggregates while promoting the reuse of materials that still retain relevant physical and mineralogical properties [2,3,4]. However, the compositional heterogeneity of these wastes, the presence of adhered mortar, and their high water absorption can affect mixture workability, modify the interfacial transition zone (ITZ), and reduce the mechanical strength of concrete [5,6]. In Mexico, several studies have mineralogically and microstructurally characterized CDW to evaluate its potential use as a raw material for new construction materials [7,8].

In parallel, the need to reduce the environmental impact associated with Portland cement production has promoted the use of supplementary mineral additions [9,10,11,12]. Among them, bentonite has attracted interest due to its mineralogical nature and predominant silica- and alumina-rich composition [11,13]. Several studies have evaluated natural or activated bentonites as partial cement replacements or as components of cementitious systems, showing that their effects depend on the type of bentonite, the incorporation level, and the characteristics of the mixture [12,14,15,16,17]. Other studies have also indicated that the incorporation of bentonite can modify the mechanical and microstructural properties of cementitious materials, as well as their pore structure and behavior under exposure to aggressive agents [16,17,18,19,20,21].

The most closely related studies show that the incorporation of bentonite into concrete has been investigated under a variety of experimental configurations. Masood et al. [18] evaluated low-calcium bentonite as a cement replacement at proportions ranging from 0 to 20%, in both natural aggregate concrete and recycled coarse aggregate concrete, considering mechanical and durability properties. Ahad et al., [16] studied mass concrete produced with natural aggregates and replacement levels of 30% bentonite, 30% fly ash, and a ternary combination containing 15% of each addition, using thermal, mechanical, mineralogical, and microstructural testing. Awan et al., [12] analyzed concrete with conventional aggregates and individual or combined replacements of bentonite and dolomite, evaluating mechanical properties and morphological characteristics by SEM.

As summarized in Table 1, these studies differ in the type of aggregate, the nature and proportion of mineral additions, the variables evaluated, and the characterization techniques employed. The study most closely related to the present work is that of Masood et al., [18]; however, their research focused on the use of recycled coarse aggregates and on mechanical and durability properties, whereas the present study incorporates both fine and coarse CDW fractions and complements the mechanical evaluation with mineralogical, morphological, and elemental characterization of the cementitious matrix.

Therefore, the contribution of the present study does not lie in proposing the combination of recycled aggregates and bentonite for the first time, but rather in evaluating a formulation consisting of fine and coarse CDW aggregates of Mexican origin and 5 wt% sodium bentonite as a partial cement replacement. The study integrates a mechanical comparison with a control mixture without bentonite, mineralogical identification by XRD, and morphological and elemental characterization by SEM-EDS, with the aim of relating composition, microstructure, and compressive strength in a concrete intended for non-structural applications.

Therefore, the objective of this study was to comparatively evaluate the mechanical behavior of concrete produced with fine and coarse CDW aggregates containing 0 and 5 wt% sodium bentonite as a partial replacement for Portland cement, and to mineralogically and microstructurally characterize the bentonite-containing system. For this purpose, XRD, SEM, EDS with elemental mapping, and compressive strength tests at 28 days were employed.

## 2. Materials and Methods

### 2.1. Materials and Characterization Techniques

Composite Portland Cement (CPC 30R), complying with NMX-C-414-ONNCE [22] and having a minimum compressive strength of 30.0 MPa (305 kg/cm^2^) at 28 days, was used. Both fine and coarse aggregates were obtained from construction and demolition waste (CDW). Qualitative inspection showed a predominance of hardened concrete and mortar fragments, with a smaller presence of ceramic and brick components.

Before use, the CDW was washed using tap water, dried at room temperature, and sieved to obtain the fine and coarse fractions. Particle size distribution was determined in accordance with NMX-C-111-ONNCCE-2018 [23], using a Ro-Tap RX-29 mechanical sieve shaker (W.S. Tyler, Mentor, OH, USA) and standardized sieves.

The sodium bentonite used was of domestic origin and had a particle size below 75 µm. As part of its physical characterization, specific gravity (2.70) was determined using a 25 mL pycnometer with a 3.00 g sample. Loss on ignition (LOI) was also determined in triplicate (10.10 ± 0.76% at 750 °C for 45 min). Mineralogical characterization was performed by XRD using an Equinox 2000 diffractometer (INEL, Artenay, France) with CoKα_1_ radiation (λ = 1.78901 Å). Crystalline phases were identified using Match! 3.0 software (Crystal impact, Bonn, Germany) and the COD Inorganics database (updated to December 2024). Morphological and elemental analysis was conducted using a JEOL JSM-IT300 scanning electron microscope (JEOL Ltd., Akishima, Tokio, Japan), coupled to an Oxford Instruments NanoAnalysis, High Wycombe, UK. The accelerating voltage was 20 kV.

### 2.2. Mix Design and Corrections for Moisture and Absorption

The mix design was developed using ACI 211.1-91 [24] for a target compressive strength of 19.61 MPa (200.0 kg/cm^2^) at 28 days. The base mixture proportions per cubic meter consisted of 360.56 kg/m^3^ of Portland cement, 181 L/m^3^ of effective water, 614.13 kg/m^3^ of fine aggregate, and 1163.01 kg/m^3^ of recycled coarse aggregate. For the control mixture (0 wt% bentonite), 360.56 kg/m^3^ of Portland cement was maintained. In the experimental formulation, 5 wt% of cement content was replaced with sodium bentonite, resulting in 342.53 kg/m^3^ of Portland cement and 18.03 kg/m^3^ of bentonite, while the total cementitious content was maintained at 360.56 kg/m^3^. The measured moisture contents were 2.04% for the fine aggregate and 1.69% for the coarse aggregate, while their water absorption values were 5.10% and 5.00%, respectively.

Because the moisture content of both fractions was lower than their absorption capacity, corrections were applied to the dosed aggregate masses, and additional water was added to compensate for the deficit relative to the saturated surface-dry (SSD) condition. The wet dosed masses were 626.66 kg/m^3^ for the fine aggregate and 1182.66 kg/m^3^ for the coarse aggregate. The net water correction was 18.79 L/m^3^ for the fine aggregate and 38.50 kg/m^3^ for the coarse aggregate, resulting in a total correction of 57.29 L/m^3^. Consequently, the total water added to the mixer was 238.29 L/m^3^, consisting of 181.00 L/m^3^ of effective design water plus 57.29 L/m^3^ to compensate for absorption. A mix design with recycled aggregates requires particular consideration of their physical and absorption characteristics, as these may modify water demand and mechanical behavior [25].

The effective water-to-cementitious material ratio (w/cm) was used because sodium bentonite partially replaced Portland cement. Based on 181.00 L/m^3^ of effective water and 360.56 kg/m^3^ of cementitious material, w/cm was 0.502 (≈0.50). When considering only the 342.53 kg/m^3^ of Portland cement, the water-to-cement ratio (w/c) was 0.528 (≈0.53). The 57.29 L/m^3^ added to compensate for absorption was not included in these ratios because it does not represent effective water available to the cement paste.

### 2.3. Specimen Preparation, Curing, and Testing

Preparation, molding, compaction, and curing of cylindrical specimens were carried out under controlled laboratory conditions using a procedure consistent with NMX-C-159-ONNCCE-2016 [26]. The concrete was placed in molds in layers and compacted by manual rodding.

For the experimental formulation containing CDW and 5 wt% sodium bentonite, six cylindrical specimens measuring 10 cm in diameter and 20 cm in height were prepared. All specimens were obtained from a single concrete batch and were produced under the same proportioning, mixing, and compaction conditions. Consequently, these specimens were considered technical replicates intended to evaluate within-batch variability and not independent experimental replicates.

Additionally, two independent control batches containing CDW and 0 wt% sodium bentonite were prepared on consecutive days. One cylindrical specimen (10 cm × 20 cm) was produced from each batch. In both batches, the total cementitious material content (360.56 kg/m^3^), w/cm (0.502), recycled aggregate quantities, and mixing, compaction, and curing conditions were maintained. The only intentional modification was the absence of bentonite and retention of the equivalent Portland cement content.

The two control batches constituted independent preparations and were intended to establish an experimental reference without bentonite and to provide an exploratory evaluation of inter-batch consistency. However, because each batch was represented by only one specimen, this comparison does not allow within-batch variability to be quantified separately or process reproducibility to be statistically demonstrated.

All specimens were demolded after 24 h and cured by immersion in clean water at 23 ± 2 °C until 28 days. Compressive strength was determined using a servo-controlled hydraulic testing machine (CONTROLS S.pA., Liscate. Italy; model 50-C79C43C04, 2000 kN capacity) located in the Civil Engineering Laboratory of the Autonomous University of the State of Hidalgo (Mineral de la Reforma, México). Tests were performed in accordance with NMX-C-083-ONNCCE-2014 [27], using an approximate loading rate of 0.25 MPa/s.

During preparation of the original batch containing 5 wt% bentonite, slump, fresh concrete density, air content, and mixture temperature were not experimentally determined. Therefore, fresh-state characterization was limited to observation of mixing, placing, and compaction. These parameters could not be determined retrospectively from the hardened specimens.

### 2.4. Statistical Analysis

For the descriptive evaluation of compressive strength, the mean, standard deviation (SD), coefficient of variation (CV), and 95% confidence interval (CI) of the mean were calculated. SD was calculated using *n* − 1 degrees of freedom, and CV was obtained as the ratio of SD to the mean, expressed as a percentage. Additionally, 95% CIs were determined using Student’s t-distribution, considering the number of observations for each formulation.

For the mixture containing 5 wt% bentonite, the six specimens corresponded to technical replicates from a single batch, whereas the two values for the control mixture corresponded to specimens from two independently prepared batches. Due to limited control observations (*n* = 2), corresponding statistics were interpreted descriptively and exploratorily.

## 3. Results

### 3.1. Physical and Granulometric Characterization

#### 3.1.1. Recycled CDW Aggregates

**Figure 1:**
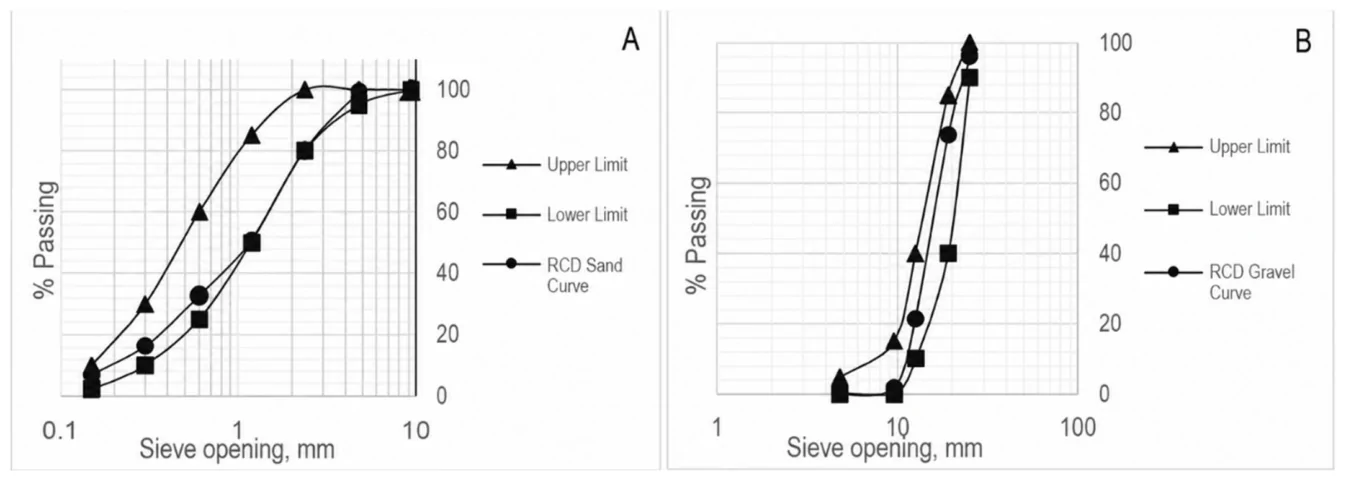
Particle size distribution curves of recycled CDW aggregates, compared with NMX-C-111-ONNCCE-2018 limits [5]: (**A**) fine fraction (CDW sand) and (**B**) coarse fraction (CDW gravel).

In addition to particle size distribution, the experimental records from the original CDW batch allowed for recovery of several physical properties. The fine fraction exhibited a relative density of 2.30, water absorption of 5.10%, initial moisture content of 2.04%, and fineness modulus of 3.18. The coarse fraction exhibited a relative density of 2.10, water absorption of 5.00%, initial moisture content of 1.69%, and bulk density of 1677 kg/m^3^. Figure 1 shows the particle size distribution curves for the fine (A) and coarse (B) fractions of recycled CDW aggregates, compared with the maximum and minimum limits established by NMX-C-111-ONNCCE-2018 [23]. In both cases, distributions fell within the ranges specified by the standard, indicating that materials met grading requirements for incorporation into the concrete mixtures. In both fractions, water absorption was higher than initial moisture content, a condition subsequently considered in water corrections applied during mix proportioning.

#### 3.1.2. Physical Characterization of Sodium Bentonite

The sodium bentonite used had a particle size below 75 µm. Physical characterization included specific gravity (2.70) determined using a 25 mL pycnometer with a 3.00 g sample and loss on ignition (LOI) determined in triplicate at 750 °C for 45 min (10.10 ± 0.76%). Results are shown in Table 2.

### 3.2. X-Ray Diffraction (XRD)

#### 3.2.1. Sodium Bentonite

**Figure 2:**
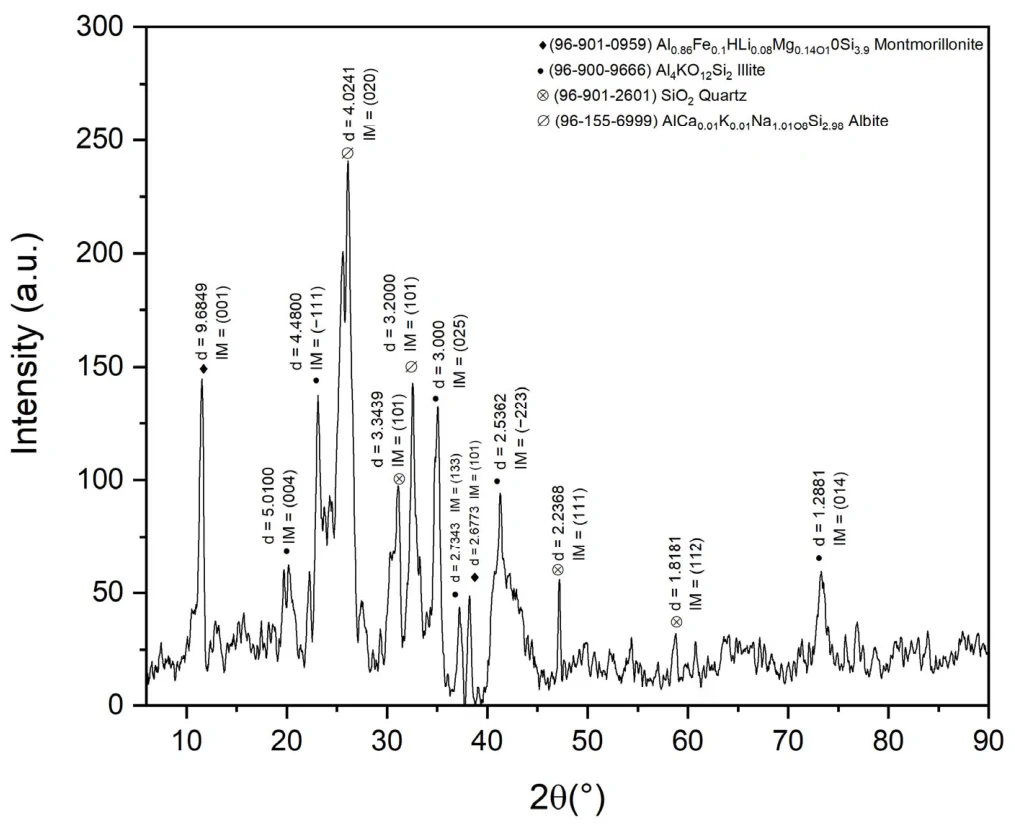
XRD pattern of sodium bentonite, showing main phases: montmorillonite, illite, quartz, and albite.

Figure 2 shows the XRD pattern of sodium bentonite, which exhibited a multiphase mineralogical composition dominated by smectite group minerals. The main phases identified were montmorillonite, illite, quartz, and albite. The most intense peaks occurred at ~2θ ≈ 20° and 36°, associated with characteristic montmorillonite reflections (PDF 96-901-0959), confirming the predominant expandable clay phase. Quartz showed its main reflection at 2θ ≈ 31.9° (d = 3.34 Å, planes (020) and (101)), whereas albite showed reflections at 2θ ≈ 25.7° and 32.2° (d = 4.024–3.200 Å, (020) and (101) planes). These results confirm the predominantly aluminosilicate nature of the bentonite (clay phases of the smectite group, mainly montmorillonite), consistent with its water absorption behavior and cation-exchange capacity.

#### 3.2.2. Construction and Demolition Waste (CDW)

**Figure 3:**
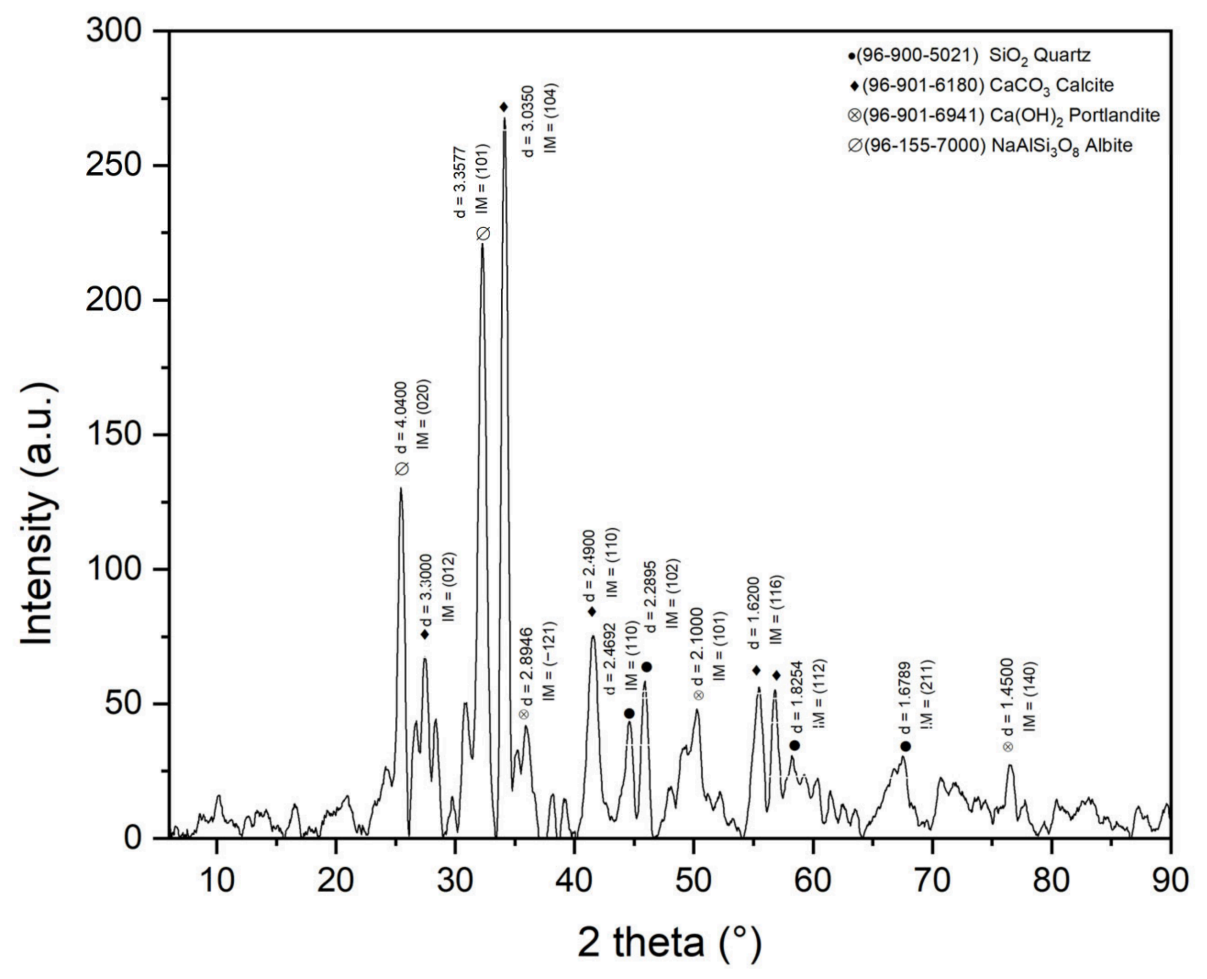
XRD pattern of CDW, showing main phases: quartz, calcite, portlandite, and albite.

The XRD pattern corresponding to CDW (Figure 3) revealed the coexistence of crystalline phases characteristic of hydrated cementitious materials and carbonated compounds. The main phases identified were quartz [SiO_2_, PDF 96-900-5021], portlandite [Ca(OH)_2_, PDF 96-901-6941], calcite [CaCO_3_, PDF 96-901-6180], and albite [NaAlSi_3_O_8_, PDF 96-155-7000]. The most intense peak was located at 2θ ≈ 34.6° (d = 3.035 Å, plane (104)), corresponding to calcite, whereas quartz exhibited a prominent reflection at 2θ ≈ 46.6° (d = 2.289 Å, plane (102)). Portlandite was identified at 2θ ≈ 36.2° and 50.2°, indicating hydration products remaining from the original cement. Albite was detected around 2θ ≈ 25.9°. Overall, this combination confirms a heterogeneous mineralogical composition associated with fragments of hardened concrete, residual mortar, and silicate components.

#### 3.2.3. Concrete with CDW and 5 wt% Sodium Bentonite

**Figure 4:**
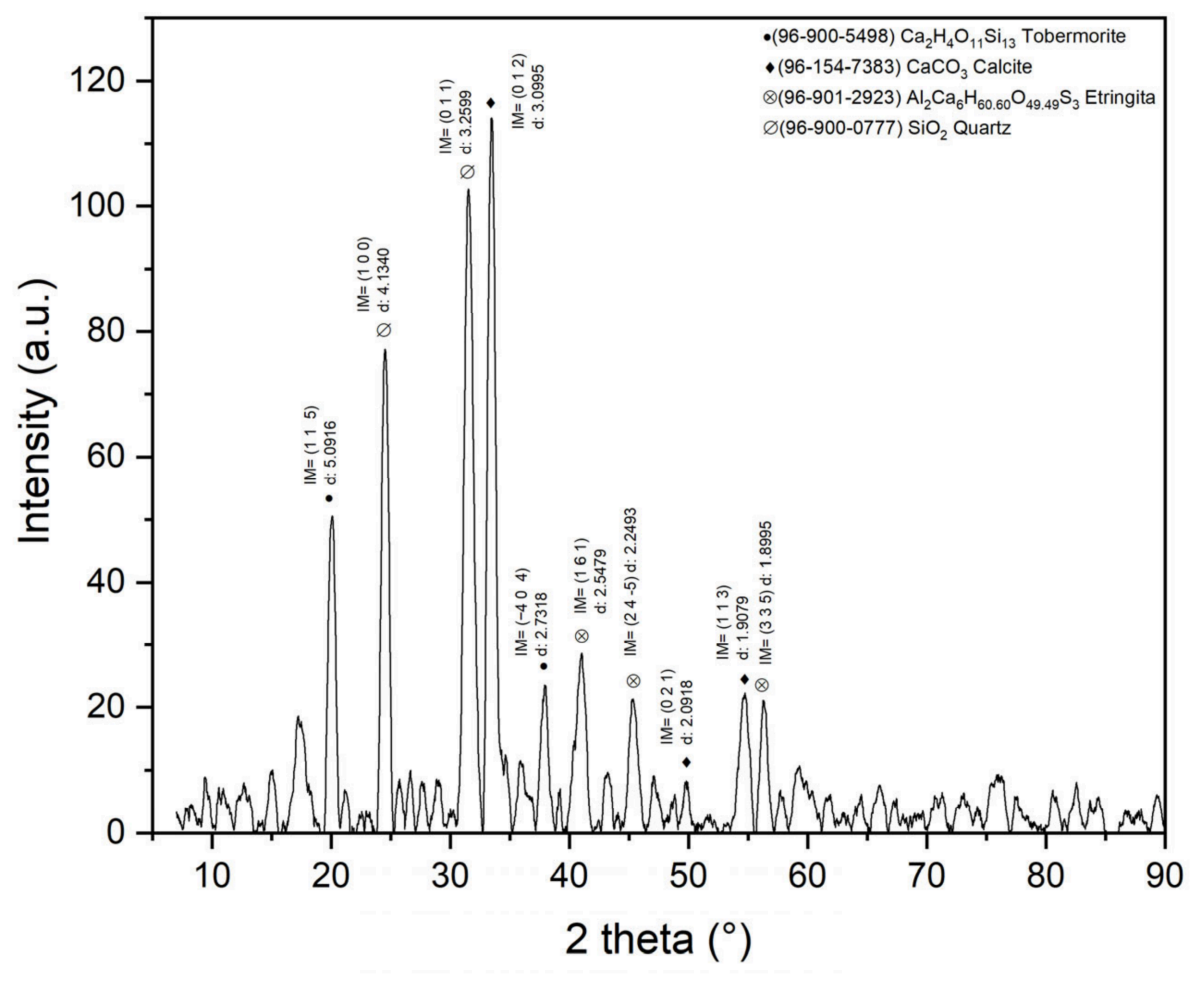
XRD pattern of concrete produced with CPC and CDW aggregates, identifying main phases: tobermorite, calcite, ettringite, and quartz.

The XRD pattern of concrete produced with Composite Portland Cement (CPC) and CDW aggregates (Figure 4) revealed crystalline phases associated with both hydration products and carbonation processes. The main phases identified were tobermorite [Ca_2_H_4_O_11_Si_3_, PFD 96-900-5498], calcite [CaCO_3_, PDF 96-154-7383], ettringite [Al_2_Ca_6_H_60_O_49.49_S_3_, PDF 96-901-2923], and quartz [SiO_2_, PDF 96-900-0777], together with signals compatible with calcium silicate hydrate (C-S-H) products.

Calcite exhibited an intense reflection around 2θ ≈ 33.3° (d = 3.09 Å, plane (102)), associated with carbonation products in the hardened matrix. Tobermorite was detected at 2θ ≈ 18.0°, while ettringite exhibited reflections around 2θ ≈ 41.1°, consistent with hydration products. Quartz was identified around 2θ ≈ 24.6°, attributable to siliceous phases in recycled aggregates. This phase combination confirms a hydrated cementitious matrix with coexisting hydrated calcium products, carbonated compounds, and residual siliceous phases.

### 3.3. Scanning Electron Microscopy (SEM)

**Figure 5:**
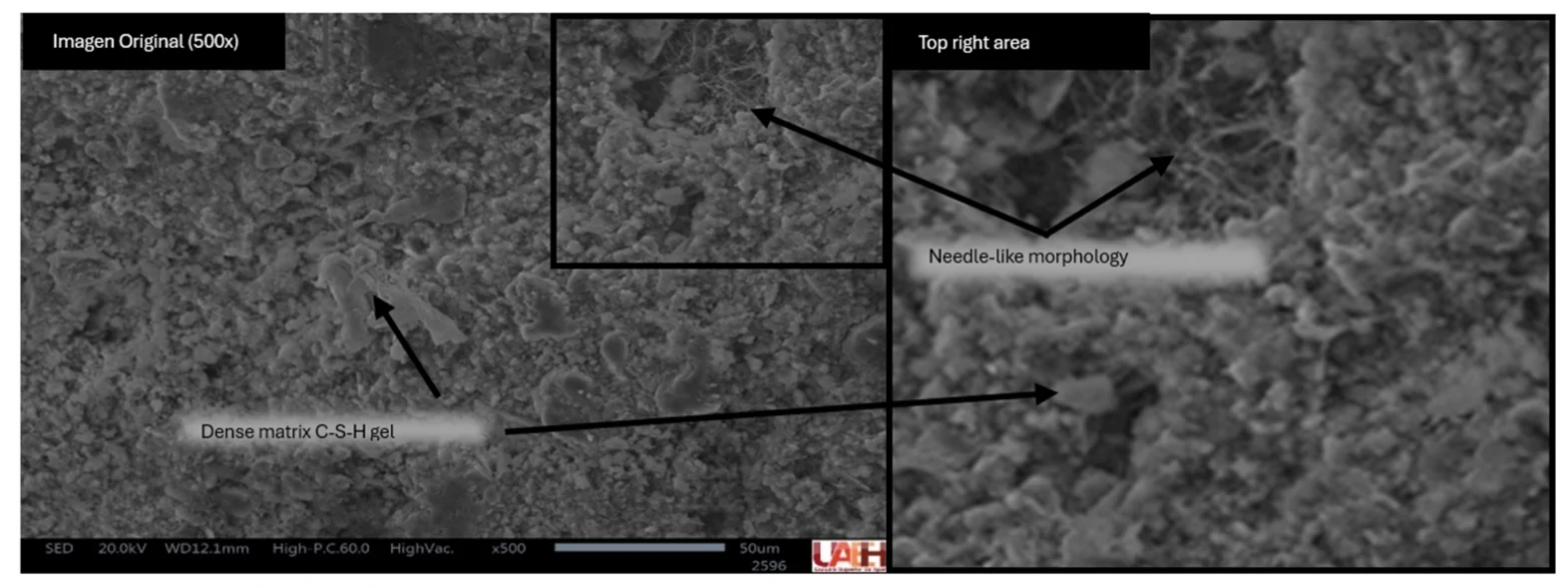
SEM micrograph of cement paste at 28 days: low-magnification overview (×500) showing heterogeneous cement matrix with dense agglomerates (possibly C-S-H). Top right area: high-magnification inset showing needle-like morphology (possible ettringite).

Figure 5 shows the SEM micrograph of concrete at 28 days (×500), together with an enlarged view of a representative area of the cementitious matrix. The low-magnification image shows a heterogeneous microstructure, with densified regions and non-uniformly distributed hydration products. The enlarged view allows two main morphologies to be distinguished: acicular structures and dense agglomerates with a globular or massive appearance.

The acicular morphologies are compatible with ettringite-type products, whereas the dense matrix and fine agglomerates may be morphologically associated with possible C-S-H gel. Overall, observations are consistent with a hydrated cementitious matrix exhibiting different morphologies associated with hydration products and heterogeneous distribution.

Because phase identification by SEM is based primarily on morphological features, assignments should be considered tentative. Therefore, microstructural interpretation was complemented with XRD and EDS results.

**Figure 6:**
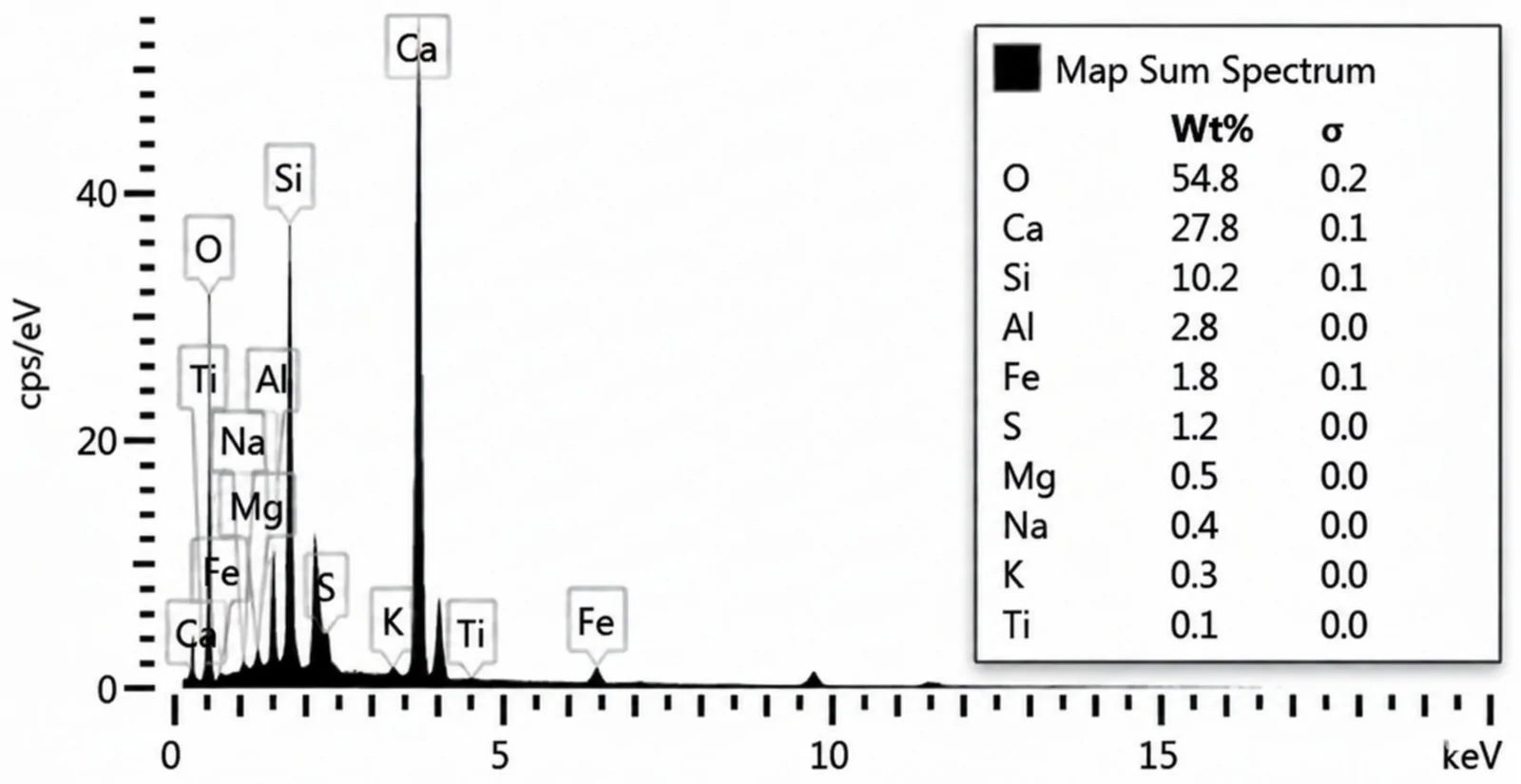
EDS spectrum of concrete with CDW and 5 wt% sodium bentonite.

The EDS spectrum (Figure 6) showed a composition dominated by oxygen (54.8 wt%) and calcium (27.8 wt%), followed by silicon (10.2 wt%) and aluminum (2.8 wt%). This composition is consistent with a hydrated cementitious matrix containing calcium- and aluminosilicate-bearing components. The presence of Si and Al may be associated with both hydration products and bentonite and recycled aggregates. Sulfur (1.2 wt%) and iron (1.8 wt%) were detected, along with smaller amounts of Mg, Na, K, and Ti (<1.0 wt%).

Because EDS provides elemental information rather than direct phase identification, these results were interpreted together with XRD and SEM. However, because the control mixture was not characterized using the same techniques, these results do not allow for a reduction in portlandite content to be established or for the formation of additional hydration products to be attributed to a pozzolanic reaction of bentonite.

**Figure 7:**
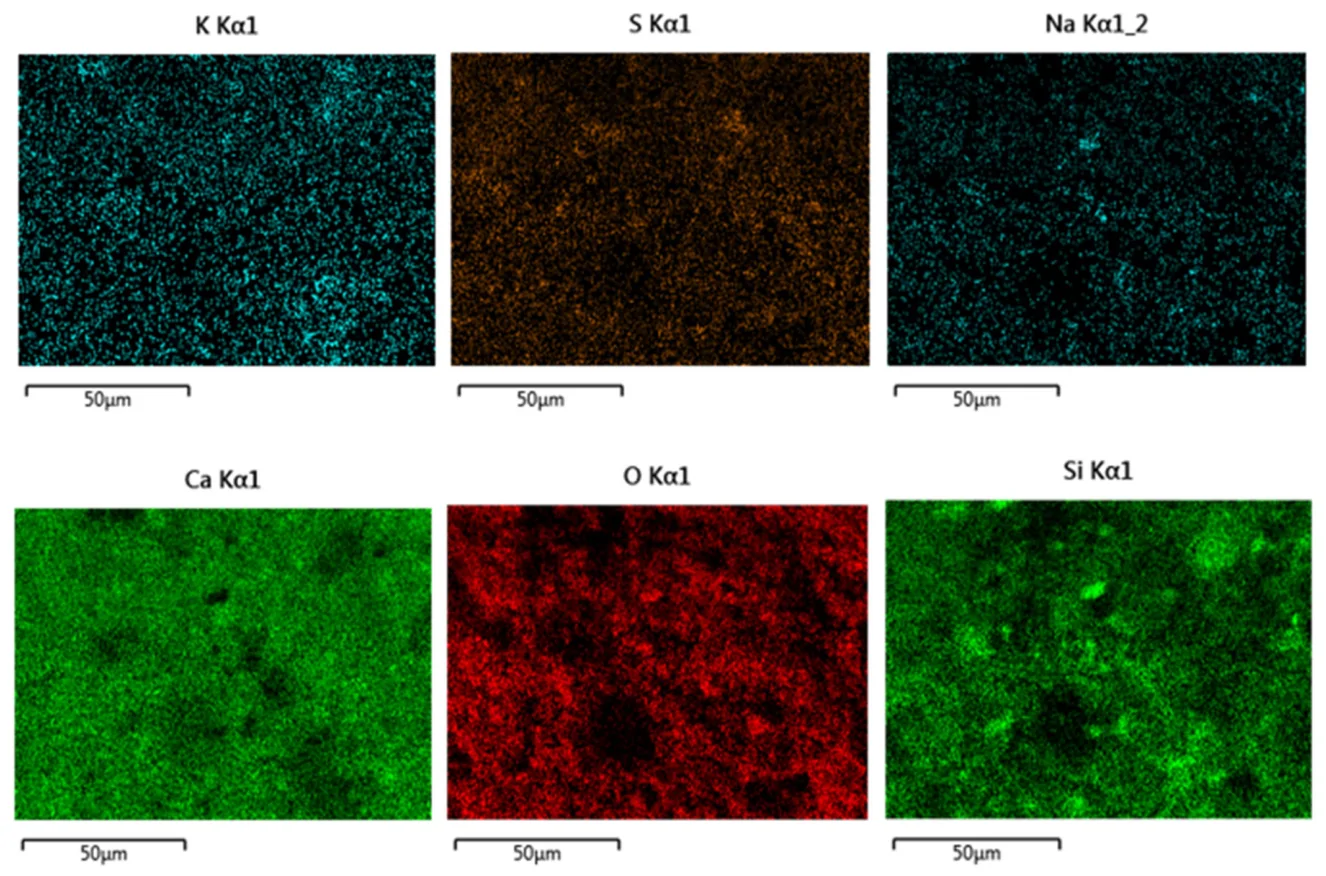

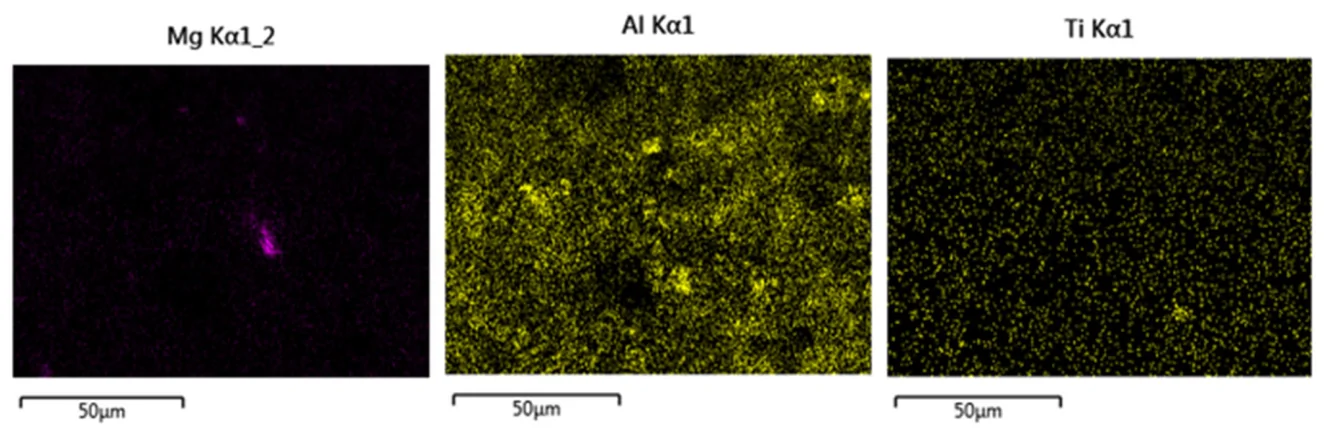
EDS elemental maps of concrete with CDW and 5 wt% sodium bentonite, showing the spatial distribution of major elements (O, Ca, Si, Al) and minor elements (Na, Mg, K, S, Ti). Colors distinguish the elements, as indicated by the labels on the individual maps.

Figure 7 shows elemental distribution mapping. The major elements O, Ca, and Si were widely distributed throughout the matrix, whereas Al and Fe were present at lower concentrations. Na, Mg, K, Ti, and S were detected in smaller proportions. The distribution is consistent with the heterogeneous nature of a cementitious matrix produced with CDW and sodium bentonite.

Overall, elemental mapping showed a relatively continuous distribution of main elements with no evidence of marked segregation at the observation scale.

### 3.4. Comparative Evaluation of Compressive Strength

**Figure 8:**
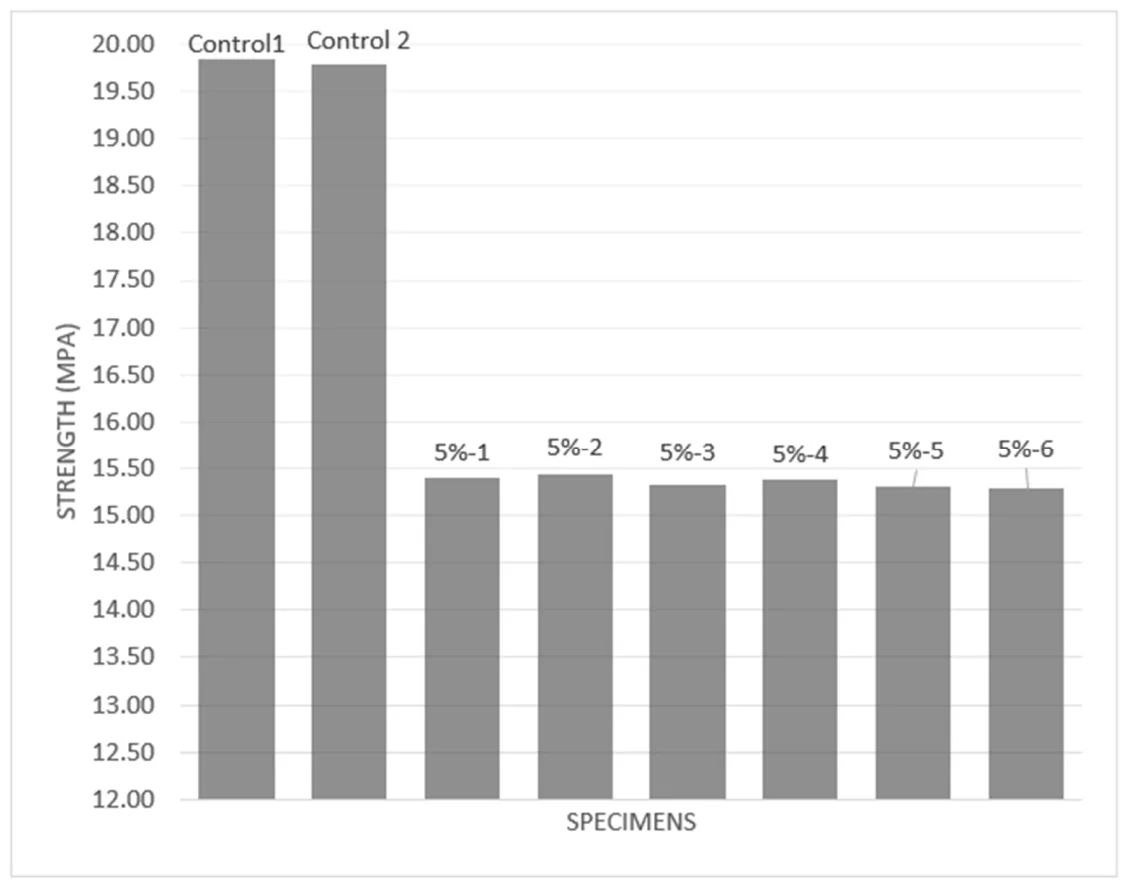
Individual 28-day compressive strength of control (0 wt% bentonite) and experimental formulation (5 wt% sodium bentonite). Target strength: 19.61 MPa (200.00 kg/cm^2^).

Figure 8 shows compressive strength results at 28 days for two formulations: the control with CDW and 0 wt% sodium bentonite, and the experimental mix with CDW and 5 wt% sodium bentonite as a partial replacement. Tests were performed per NMX-C-083-ONNCCE-2014 [27], using 10 cm × 20 cm cylinders. The two control specimens, RCc-RMn-10 and RCc-RMn-15, were obtained from independent batches prepared on different days under identical proportioning, mixing, compaction, curing, and testing conditions. The obtained compressive strengths were 19.833 (202.24 kg/cm^2^) and 19.788 MPa (201.78 kg/cm^2^), respectively, with a mean value of 19.810 MPa (202.00 kg/cm^2^). The absolute difference was 0.045 MPa (0.46 kg/cm^2^), corresponding to a relative difference of 0.228% versus the mean.

The two control batches showed an SD of 0.032 MPa (0.32 kg/cm^2^) and a CV of 0.161%. The 95% CI ranged from 19.524 to 20.097 MPa (199.08–204.93 kg/cm^2^). The relative difference of only 0.228% indicates closeness between the two observed values.

Six specimens from the 5 wt% sodium bentonite mixture exhibited strengths of 15.406 (157.09 kg/cm^2^), 15.434 (157.38 kg/cm^2^), 15.322 (156.24 kg/cm^2^), 15.371 (156.74 kg/cm^2^), 15.302 (156.03 kg/cm^2^), and 15.284 MPa (155.85 kg/cm^2^). The mean was 15.353 MPa (156.55 kg/cm^2^), with an SD of 0.060 MPa (0.61 kg/cm^2^) and CV of 0.391%. The 95% CI ranged from 15.290 to 15.416 MPa (155.91–157.19 kg/cm^2^). These six specimens were technical replicates from a single batch; the low SD and CV indicate low within-batch dispersion.

The target strength per ACI 211.1-91 [24] was 19.61 MPa (200.0 kg/cm^2^) at 28 days. The mean control value was 19.810 MPa (101.02% of design), whereas the 5 wt% bentonite mixture reached 15.353 MPa (78.29% of design). Direct comparison showed a 4.457 MPa (45.45 kg/cm^2^) lower mean, representing a 22.50% reduction at 28 days.

Table 3 summarizes the statistical results, showing low dispersion for both formulations. The control showed a CV of 0.161%, whereas 5 wt% bentonite showed a CV of 0.391%. The confidence intervals do not overlap. However, due to limited control specimens (*n* = 2) and the technical nature of bentonite replicates (single batch), the confidence intervals are presented descriptively, and the results should be interpreted descriptively.

## 4. Discussion

The results allow for a comparison of the mechanical behavior of mixtures produced with recycled construction and demolition waste (CDW) aggregates in the presence and absence of sodium bentonite. Characterization by XRD, SEM, and EDS was performed only on the mixture containing 5 wt% bentonite at 28 days. Therefore, these analyses enabled the mineralogical composition, morphologies, and elemental distribution of its hardened matrix to be described, but did not enable direct microstructural differences relative to the control mixture to be established or changes over time to be assessed. Consequently, the identified phases and morphologies cannot be attributed exclusively to bentonite.

XRD analysis revealed the presence of calcite, ettringite, quartz, and signals assigned to phases consistent with calcium silicate hydrate (C-S-H) products. Complementarily, SEM observations revealed a heterogeneous microstructure, with acicular morphologies and regions exhibiting a densified appearance. These features are consistent with hydration products commonly found in cementitious matrices; however, their identification should be regarded as tentative, since morphology alone does not enable a phase to be unequivocally identified.

EDS analysis showed a composition dominated by O, Ca, and Si, with Al and other minor elements also present, consistent with a hydrated cement matrix containing aluminosilicate components derived from both bentonite and CDW. Elemental mapping showed distribution of Ca, Si, and Al within the analyzed region, with no evidence of marked segregation at the observation scale. Nevertheless, these results alone do not demonstrate either the occurrence or the extent of a pozzolanic reaction. Confirmation of a reduction in portlandite content or additional formation of hydration products would require quantitative comparison with the control mixture using XRD, thermal analysis, or other complementary techniques. Therefore, the possible contribution of bentonite to matrix characteristics should be interpreted as a hypothesis consistent with the results rather than as a fully demonstrated causal effect.

The inclusion of a control mixture provided a direct experimental reference for assessing the mechanical effect of partially replacing Portland cement with sodium bentonite. The control specimens RCc-RMn-10 and RCc-RMn-15 reached 19.833 MPa (202.24 kg/cm^2^) and 19.788 MPa (201.78 kg/cm^2^), respectively, with a mean of 19.810 MPa (202.00 kg/cm^2^). In contrast, the mixture containing 5 wt% bentonite exhibited a mean of 15.353 MPa (156.55 kg/cm^2^). The difference was 4.457 MPa (45.45 kg/cm^2^), corresponding to a 22.50% reduction relative to the control. These results indicate that, under the evaluated conditions, replacing 5 wt% of Portland cement with sodium bentonite was associated with a decrease in 28-day compressive strength.

The control group contains only two observations, with one specimen representing each independently prepared batch, whereas the six specimens in the bentonite group are technical replicates obtained from a single batch. Consequently, confidence intervals are presented descriptively and non-overlapping confidence intervals should not be interpreted as evidence of a statistically significant difference between the formulations. The two observed control values were close to each other (relative difference 0.228%, 0.045 MPa (0.46 kg/cm^2^)), providing a preliminary experimental reference. The CV of 0.161% reflects low dispersion between these two values, but due to limited control observations (*n* = 2) and the technical nature of bentonite replicates, the comparison remains descriptive and does not allow for formal inference of statistical significance.

The comparative behavior may be related to the simultaneous action of several factors. Partial replacement of Portland cement with bentonite reduces the amount of cement available for hydration and may result in a dilution effect. Likewise, incorporation of fine bentonite particles may modify water demand and workability. Conversely, these particles may contribute to the distribution of solids through a filler effect. The relative magnitude of these mechanisms depends on mixture proportions, effective water availability, compaction, and the intrinsic characteristics of bentonite.

Physical characterization of bentonite showed a specific gravity of 2.70 and an LOI of 10.10 ± 0.76% at 750 °C for 45 min (*n* = 3). This parameter provides additional information on the material used but does not enable the observed mass loss to be attributed to a specific mechanism, because no complementary thermal or chemical analyses (TGA, XRF) were performed.

Interpretation of these effects is limited by the absence of fresh concrete measurements during preparation of the original 5 wt% bentonite batch. Slump, fresh density, air content, and mixture temperature were not determined. This omission is relevant because the high absorption of CDW materials and the presence of bentonite may affect consistency, compactability, and void content. Therefore, it is not possible to fully distinguish the effect of partial cement replacement from potential unquantified variations in workability and compaction.

The physical properties of the recycled aggregates showed that, for both fractions, water absorption (5.10% fine, 5.00% coarse) was higher than initial moisture content (2.04% fine, 1.69% coarse). This condition justified a water correction of 57.29 L/m^3^ applied during proportioning and represents a relevant factor in maintaining effective water availability. Differences in relative density (2.30 fine, 2.10 coarse) and the particle size characteristics reflect the heterogeneous nature of the CDW materials used.

The composition of CDW materials was qualitatively characterized by a predominance of hardened concrete and mortar fragments, with smaller amounts of ceramic and brick components. These characteristics may have influenced the bond between the recycled aggregates and cementitious matrix and, consequently, stress transfer during compression. However, because constituent composition, adhered mortar content, contaminant materials, and resistance to wear or crushing were not quantified, the independent contribution of each variable cannot be established.

The results should also be considered in relation to the most closely related previous studies. Masood et al. [18] investigated concrete containing recycled aggregates and 0–20% replacement of cement with low-calcium bentonite, considering mechanical and durability properties. Although this represents the most closely related previous work, its approach focused primarily on recycled coarse aggregate and on mechanical and durability performance, whereas the present study simultaneously incorporated fine and coarse CDW fractions and included mineralogical, morphological, and elemental characterizations of a matrix containing 5 wt% bentonite.

In contrast, Ahad et al. [16] investigated binary and ternary mixtures of bentonite and fly ash in concrete produced with conventional aggregates, using XRD, SEM, thermal analysis, and mechanical testing. Differences in aggregate type, replacement proportions, and the combination of supplementary materials limit direct comparison. Nevertheless, this work highlights the importance of jointly considering hydration products, microstructural characteristics, and mechanical performance when aluminosilicate materials are incorporated.

Awan et al. [12] evaluated bentonite and dolomite replacement levels ranging from 2.5 to 10 wt% in concrete produced with conventional aggregates through mechanical testing and SEM. In contrast to that study, the present investigation used sodium bentonite at 5 wt% and replaced conventional fine and coarse aggregates with fractions derived from CDW materials. These differences are relevant because the mechanical response of a bentonite-containing mixture may be influenced by absorption of recycled aggregates, presence of residual mortar, quality of ITZ, and effective availability of water. Therefore, strength values reported for systems containing natural aggregates cannot be directly extrapolated to concrete produced with CDW materials.

The mixture containing 5 wt% bentonite achieved 15.353 MPa (156.55 kg/cm^2^), equivalent to 78.29% of the target 19.61 MPa (200.0 kg/cm^2^). In contrast, the control mixture reached 19.810 MPa (202.00 kg/cm^2^), corresponding to 101.02% of the design value. This comparison shows that the control formulation achieved the strength specified by ACI 211.1-91, whereas partial replacement with 5 wt% bentonite resulted in a 22.50% reduction relative to the control. Six bentonite-containing specimens exhibited an SD of 0.060 MPa (0.61 kg/cm^2^) and CV of 0.391%, indicating low dispersion among technical replicates from the same batch.

From a practical perspective, the compressive strength obtained for the 5 wt% bentonite mixture falls within the range used for applications with low mechanical strength requirements. The 2025 General Unit Price Schedule (Tabulador) of the Government of Mexico City includes hydraulic concrete blinding layers with ~9.81 MPa (100.0 kg/cm^2^), as well as slabs and floors with ~14.71 MPa (150.0 kg/cm^2^) and 19.61 MPa (200.0 kg/cm^2^) [28]. In this context, the value achieved suggests potential use in non-structural applications. However, final selection should be based on the particular project specifications and assessment of additional properties related to durability, absorption, permeability, and service performance.

The scope of the results is subject to several limitations. Only one sodium bentonite replacement level (5 wt%) and one primary testing age (28 days) were evaluated. Furthermore, although two independent control batches were included, each was represented by only one specimen. Therefore, comparison provides a preliminary experimental reference but does not constitute a complete statistical assessment of inter-batch variability.

Characterization of recycled aggregates was limited to information available in original experimental records. Although results were available for the particle size distribution, relative density, water absorption, initial moisture content, fineness modulus (3.18) of the fine aggregate, and bulk density (1677 kg/m^3^) of the coarse aggregate, the adhered mortar content, resistance to wear or crushing, gravimetric composition, and quantitative contaminant content were not determined. Because a representative sample of the original CDW batch was not retained, these properties could not be evaluated subsequently.

Hardened concrete properties such as water absorption, permeability, porosity, shrinkage, and durability against aggressive agents were also not determined. Furthermore, the absence of XRD, SEM, and EDS characterization of the control mixture prevents the specific effect of bentonite on the observed phases and morphologies from being isolated. Consequently, based on the available results, it cannot be concluded that bentonite reduced porosity, significantly decreased portlandite content, or improved the durability.

Future studies should consider a larger number of independent batches and multiple specimens per batch, as well as different bentonite replacement levels and curing ages beyond 28 days. Parallel characterization of control and bentonite-containing formulations using XRD, SEM, EDS, thermal analysis, and other complementary quantitative techniques will be necessary.

Experimental characterization should include fresh concrete properties, particularly slump, density, air content, and temperature. Furthermore, a more comprehensive evaluation of recycled aggregates should be conducted, including quantitative constituent composition, adhered mortar content, presence of contaminants, and resistance to wear or crushing. The inclusion of water absorption, permeability, porosity, and durability tests would help determine whether observed microstructural characteristics translate into adequate functional performance.

The low dispersion observed among six specimens of 5 wt% bentonite formulation may be related, among other factors, to their origin from the same concrete batch. Because specimens were produced from the same material preparation, they were subjected to similar conditions regarding aggregate distribution, water availability, mixing, and compaction. Use of the same proportions of CDW materials may have favored relatively uniform distribution of constituents within the batch and contributed to consistency of results. This interpretation should be regarded as a possible contributing factor, since the aggregates contained in each individual specimen were not characterized separately.

Overall, the results show that a mixture incorporating fine and coarse CDW aggregates and 5 wt% sodium bentonite exhibited consistent compressive strength among specimens from the single experimental batch. Its cementitious matrix contained hydrated and carbonated phases at 28 days. Comparison with the control mixture established that the formulation containing 5 wt% bentonite exhibited a 22.50% reduction (4.457 MPa; 45.45 kg/cm^2^) in 28-day compressive strength relative to concrete without bentonite.

## 5. Conclusions

The comparison between the control mixture produced with CDW and 0 wt% sodium bentonite and the experimental mixture containing CDW and 5 wt% sodium bentonite allowed the mechanical behavior associated with partial replacement of Portland cement to be evaluated. The control specimens reached 19.833 MPa (202.24 kg/cm^2^) and 19.788 MPa (201.78 kg/cm^2^), with a mean of 19.810 MPa (202.00 kg/cm^2^; 101.02% of target), whereas the formulation containing 5 wt% bentonite exhibited a mean of 15.353 MPa (156.55 kg/cm^2^; 78.29% of target). Consequently, the 5 wt% bentonite formulation exhibited a 22.50% lower (4.457 MPa; 45.45 kg/cm^2^) 28-day compressive strength than the control mixture.

The control mixture achieved approximately 101.02% of the target strength of 19.61 MPa (200.0 kg/cm^2^) established using ACI 211.1-91. In contrast, the mixture containing 5 wt% bentonite achieved 78.29% of the design value. The two control results showed close agreement (relative difference 0.228%), with a CV of 0.161% (SD 0.032 MPa; 0.32 kg/cm^2^), whereas the six specimens of the bentonite-containing formulation exhibited a CV of 0.391% (SD 0.060 MPa; 0.61 kg/cm^2^), indicating low dispersion within the experimental batch (95% CI 15.290–15.416 MPa).

XRD, SEM, and EDS characterization of the 5 wt% bentonite formulation identified calcite, ettringite, quartz, and signals consistent with calcium silicate hydrate (C-S-H) products, together with acicular morphologies and dense agglomerates and an elemental distribution dominated by Ca, Si, and Al. However, because the control mixture was not characterized using the same techniques, observed phases and microstructural characteristics cannot be attributed exclusively to incorporation of sodium bentonite, nor can a pozzolanic effect be confirmed.

Although the 5 wt% bentonite formulation did not achieve the target strength of 19.61 MPa (200.0 kg/cm^2^), its mean strength of 15.353 MPa (156.55 kg/cm^2^) falls within the 9.81–19.61 MPa (100.0–200.0 kg/cm^2^) bracket specified in the 2025 General Unit Price Schedule (Tabulador) of the Government of Mexico City for hydraulic concrete blinding layers, with ~9.81 MPa (100.0 kg/cm^2^), and for slabs and floors with ~14.71–19.61 MPa (150.0–200.00 kg/cm^2^). In this context, the formulation may be considered for non-structural applications with low strength requirements, provided that suitability is verified against project-specific design requirements and complementary durability testing.

The scope of the results is limited by the small number of control specimens (*n* = 2, independent batches, one specimen each), use of a single bentonite replacement level (5 wt%), and primary evaluation at 28 days. In addition, six specimens containing 5 wt% bentonite were technical replicates from a single batch; therefore, low dispersion primarily represents within-batch variability. Future studies should include a larger number of independent batches and specimens per batch, different replacement levels, additional curing ages, characterization of fresh concrete properties (slump, density, air content, temperature), and equivalent microstructural characterization of both control and experimental formulations, including quantitative XRF and thermal analysis.

## Figures and Tables

**Table 1:** Comparison of studies related to the use of bentonite and recycled aggregates in concrete.

Reference	Aggregate Type	Addition and Replacement	Variables and Methods	Main Result	Difference Compared to Present Study
Masood et al., [18]	Recycled coarse aggregate; comparison with natural aggregate	Low-calcium bentonite; 0, 5, 10, 15, and 20 wt% cement replacement	Workability, fresh density, air content, compressive strength and splitting tensile strength, water absorption, chloride migration, and acid attack	Bentonite mainly improved later-age strength and the durability of recycled aggregate concrete	Most closely related study; however, it focuses on recycled coarse aggregates and durability, whereas present study uses both fine and coarse CDW fractions and complements mechanical evaluation with mineralogical, morphological, and elemental characterization
Ahad et al., [16]	Crushed natural aggregates in mass concrete	30 wt% bentonite; 30 wt% fly ash; and ternary blend 15 wt% bentonite and 15 wt% fly ash	Thermal evolution, compressive strength, ultrasonic pulse velocity, DTA, TGA, XRD, and SEM	Bentonite reduced temperature rise in mass concrete and modified evolution of hydration products; mechanical effect depended on the formulation	Focuses on mass concrete with natural aggregates and high replacement levels, whereas present study uses 5 wt% sodium bentonite and CDW
Awan et al., [12]	Conventional fine and coarse aggregates	Bentonite and dolomite individually at 2.5, 5, and 10%; combined 2.5% + 2.5% and 5% + 5%	Compressive strength, flexural strength, and splitting tensile strength; SEM	Bentonite individually increased compressive strength; at 5%, 8.94% increase at 28 days. Higher combined contents reduced strength	Evaluates natural aggregates and bentonite–dolomite interaction, without CDW or XRD and EDS characterization
Present study	Fine and coarse CDW fractions of Mexican origin	Uncalcined sodium bentonite; control 0 wt% and experimental 5 wt% cement replacement	Compressive strength at 28 days; XRD, SEM, EDS, and elemental mapping	Relationship between mineralogical, compositional, and microstructural characteristics, and mechanical performance of CDW concrete intended for non-structural applications	Integrates both CDW fractions, mechanical comparison with control, and mineralogical, morphological, and elemental characterization

**Table 2:** Physical properties of sodium bentonite.

Property	Test Condition	Result
Particle size	Material used	<75 µm
Specific gravity	25 mL pycnometer; 3.00 g sample	2.70
Loss on ignition	750 °C; 45 min; *n* = 3	10.10 ± 0.76%

**Table 3:** Statistical results of the compressive strength at 28 days.

Mixture	*n*	Mean, MPa	SD, MPa	CV (%)	95% CI, MPa
Control, 0 wt% bentonite	2 (2 independent batches)	19.810	0.032	0.161	19.524–20.097
Experimental, 5 wt% bentonite	6 (technical replicates, single batch)	15.353	0.060	0.391	15.290–15.416

## Data Availability

The original contributions presented in this study are included in the article. Further inquiries can be directed to the corresponding authors.
